# The prognostic value of the preoperative albumin-to-fibrinogen ratio in patients with intrahepatic cholangiocarcinoma: a multicenter retrospective propensity score matching analysis

**DOI:** 10.3389/fonc.2025.1633488

**Published:** 2025-11-03

**Authors:** Yakai Yang, Junjie Kong, Tao Wang, Min Ji, Guangbing Li, Jun Liu

**Affiliations:** ^1^ Department of Hepatobiliary Surgery, Shandong Provincial Hospital, Cheeloo College of Medicine, Shandong University, Jinan, Shandong, China; ^2^ Department of Hepatobiliary Surgery, Shandong Provincial Hospital Affiliated to Shandong First Medical University, Jinan, Shandong, China; ^3^ Department of Liver Surgery, West China Hospital, Sichuan University, Chengdu, Sichuan, China; ^4^ Department of Hepatobiliary and Pancreatic Surgery, First Hospital Affiliated to Zhengzhou University, Zhengzhou, Henan, China

**Keywords:** intrahepatic cholangiocarcinoma, albumin-to-fibrinogen ratio, prognosis, propensity score matching, nomogram

## Abstract

**Background:**

This study evaluated the prognostic role of the albumin-to-fibrinogen ratio (AFR) in patients with intrahepatic cholangiocarcinoma (ICC) after curative liver resection.

**Methods:**

Retrospectively analyzed the clinicopathological information of ICC patients and stratified them into two groups by AFR (8.71). A 1:3 propensity score matching (PSM) analysis was used to eliminate possible biases. Kaplan-Meier method was used for survival analysis. Independent prognostic factors for overall survival (OS) and disease-free survival (DFS) were analyzed using Cox regression analysis, and based on which two nomograms were constructed. The concordance index (C-index), decision curve analysis (DCA), calibration curve, and receiver operating characteristic (ROC) curve were used to validate the nomograms.

**Results:**

559 patients were included and were divided into low- and high-AFR groups, respectively. High-AFR group had better prognosis. The multivariate analysis revealed that AFR was an independent prognostic factor for both OS (hazard ratio [HR] 0.393, *P* < 0.001) and DFS (HR 0.538, *P* < 0.001). Two nomograms were established to predict OS and DFS, and demonstrated high predictive accuracy and clinical utility. Furthermore, ROC curves demonstrated the high predictive power of the nomogram for survival in ICC patients.

**Conclusions:**

Preoperative AFR was an independent prognostic factor for postoperative OS and DFS in ICC patients, and AFR-based nomograms effectively predict postoperative survival outcomes.

## 1 Introduction

Intrahepatic cholangiocarcinoma (ICC) is the second most common primary liver cancer, with increasing incidence and mortality worldwide (1, 2). ICC is a malignant tumor originating from the proximal secondary bile ducts within the liver parenchyma, often associated with primary sclerosing cholangitis (PSC), liver fluke infection and viral hepatitis are risk factors of ICC (3–5). Surgical resection remains the primary treatment. However, owing to its insidious onset and high invasiveness, liver resection only provides 5-year overall survival ranging from 20% to 40% (6–8). Therefore, identifying prognostic factors is crucial for improving clinical outcomes.

The TNM staging system established by the American Joint Committee on Cancer (AJCC) is considered the gold standard for ICC prognostication (9), but its limitations, especially in T staging (10, 11), underscore the necessity for novel prognostic markers. Emerging evidence highlights the role of malnutrition, inflammation and coagulation in the occurrence and progression of tumors (12–14). Albumin (ALB) is a widely utilized clinical indicator for assessing nutritional status, and previous study have indicated that decreased preoperative serum albumin levels are associated with poor postoperative outcomes in ICC patients (15). In addition, elevated fibrinogen (FIB) may contribute to hypercoagulability and tumor progression across various malignancies, with high fibrinogen linked to poor prognosis in cholangiocarcinoma patients (16). However, not all patients exhibit both nutritional deficiencies and coagulopathy. In recent years, the albumin-to-fibrinogen ratio (AFR), a novel composite biomarker, has been reported to be associated with the prognosis of various cancers, including gallbladder cancer, pancreatic cancer and hepatocellular carcinoma (17–19). However, there are currently no studies investigating the prognostic significance of AFR in ICC patients undergoing surgery.

In this study, we evaluated the prognostic value of preoperative AFR in ICC patients after curative liver resection and developed nomograms to predict their overall survival (OS) and disease-free survival (DFS).

## 2 Materials and methods

### 2.1 Patient selection

A retrospective analysis was performed on the clinical and follow-up data of patients who underwent surgery for ICC between July 2009 and October 2022 at Shandong Provincial Hospital Affiliated to Shandong University, West China Hospital of Sichuan University, and First Hospital Affiliated to Zhengzhou University. This study was approved by the Medical Ethics Committees of Shandong Provincial Hospital Affiliated to Shandong University, West China Hospital of Sichuan University, and First Hospital Affiliated to Zhengzhou University. Relevant data can be used in clinical research on the premise of anonymity, and all patients provided informed consent. The inclusion criteria were: (1) Over 18 years old; (2) Underwent surgical resection; (3) Postoperative pathological diagnosis of ICC; (4) Not received preoperative neoadjuvant chemotherapy or radiotherapy. The exclusion criteria included: (1) Presence of distant metastasis; (2) History of other malignant tumors; (3) Severe organ dysfunction; (4) Data missing or lost to follow-up.

### 2.2 Data collection

The demographic data included age, sex, height, weight, alcohol consumption, and the presence of diabetes and hepatitis. Blood samples were collected 3 to 7 days prior to surgery, encompassing routine blood tests, liver function tests, blood biochemical tests, alpha-fetoprotein (AFP), carbohydrate antigen 199 (CA199), carbohydrate antigen 125 (CA125) and carcinoembryonic antigen (CEA). Postoperative pathological features included tumor number, tumor size, tumor differentiation, satellite lesions, microinvasive carcinoma (MCI), microvascular invasion (MVI), perineural invasion, liver capsule invasion, lymph node metastasis and TNM staging. Neutrophil-to-lymphocyte ratio (NLR) = absolute neutrophil count/absolute lymphocyte count; platelet-to-lymphocyte ratio (PLR) = absolute platelet count/absolute lymphocyte count. TNM staging was evaluated according to the AJCC 8th edition (20). For AFR determination, AFR = absolute albumin count/absolute fibrinogen count. Albumin was measured using the bromocresol green (BCG) assay with the certified reference material ERM-DA470k/IFCC from the JRC’s Reference Materials and Measurements group for quality control, while fibrinogen was quantified via the Clauss method from citrate-anticoagulated plasma processed within 1 hour using the World Health Organization (WHO) Fibrinogen Plasma 3rd International Standard (code 09/264) for quality control (21–23).

### 2.3 Follow up

**Figure 1:**
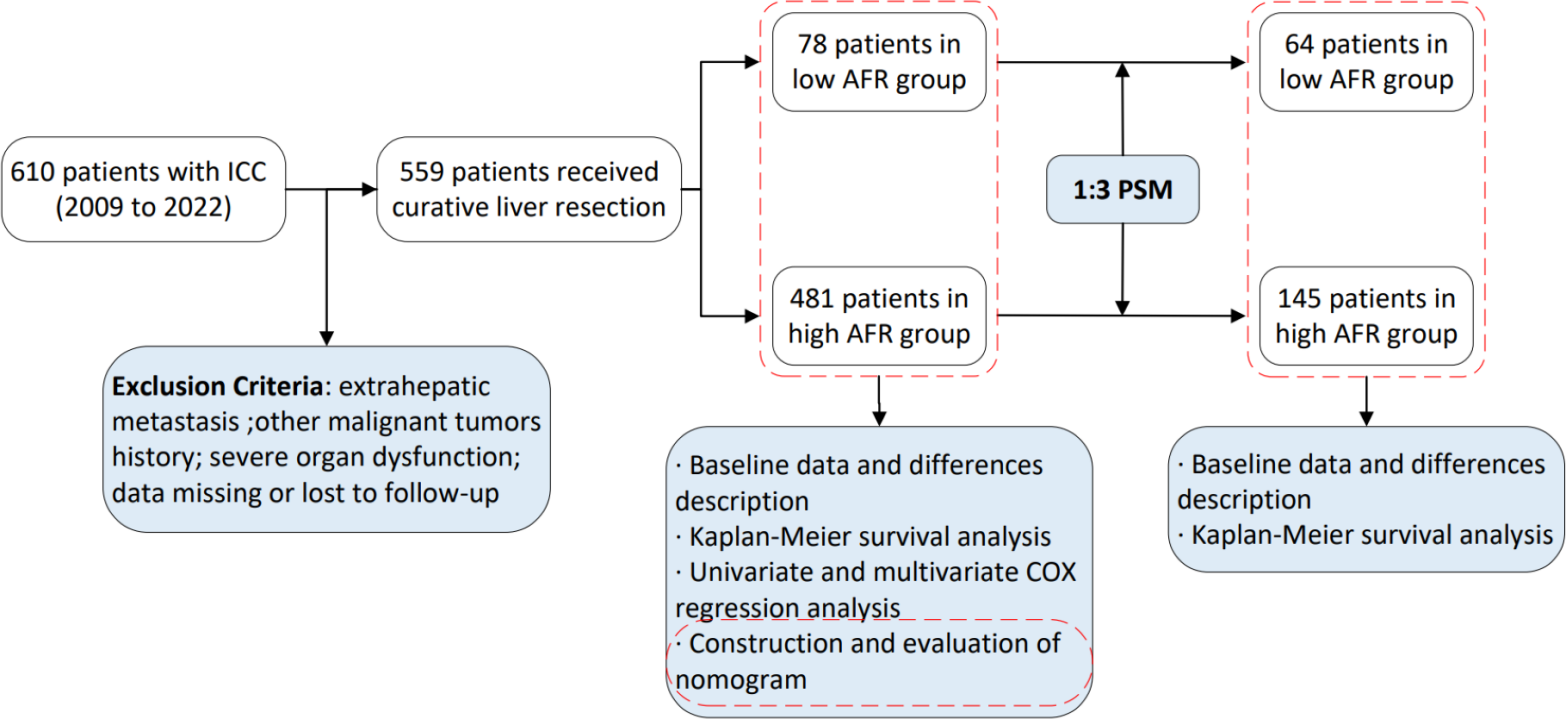
Flow chart of patient selection and study workflow.

Patients were followed up one month after discharge and subsequently every three months thereafter until death or loss to follow-up. During follow-up, serological tests and enhanced computed tomography or magnetic resonance imaging were performed, with tumor recurrence assessed based on these tests. The study endpoints included OS and DFS. OS was defined as the interval from the start of surgery to the date of death or last follow-up, while DFS was defined as the interval from the start of surgery to the date of objective tumor progression, death or last follow-up. All patients were followed up until August 31, 2023. The flow chart depicting patient selection and study work is presented in **Figure 1**.

### 2.4 Statistical analysis

**Figure 2:**
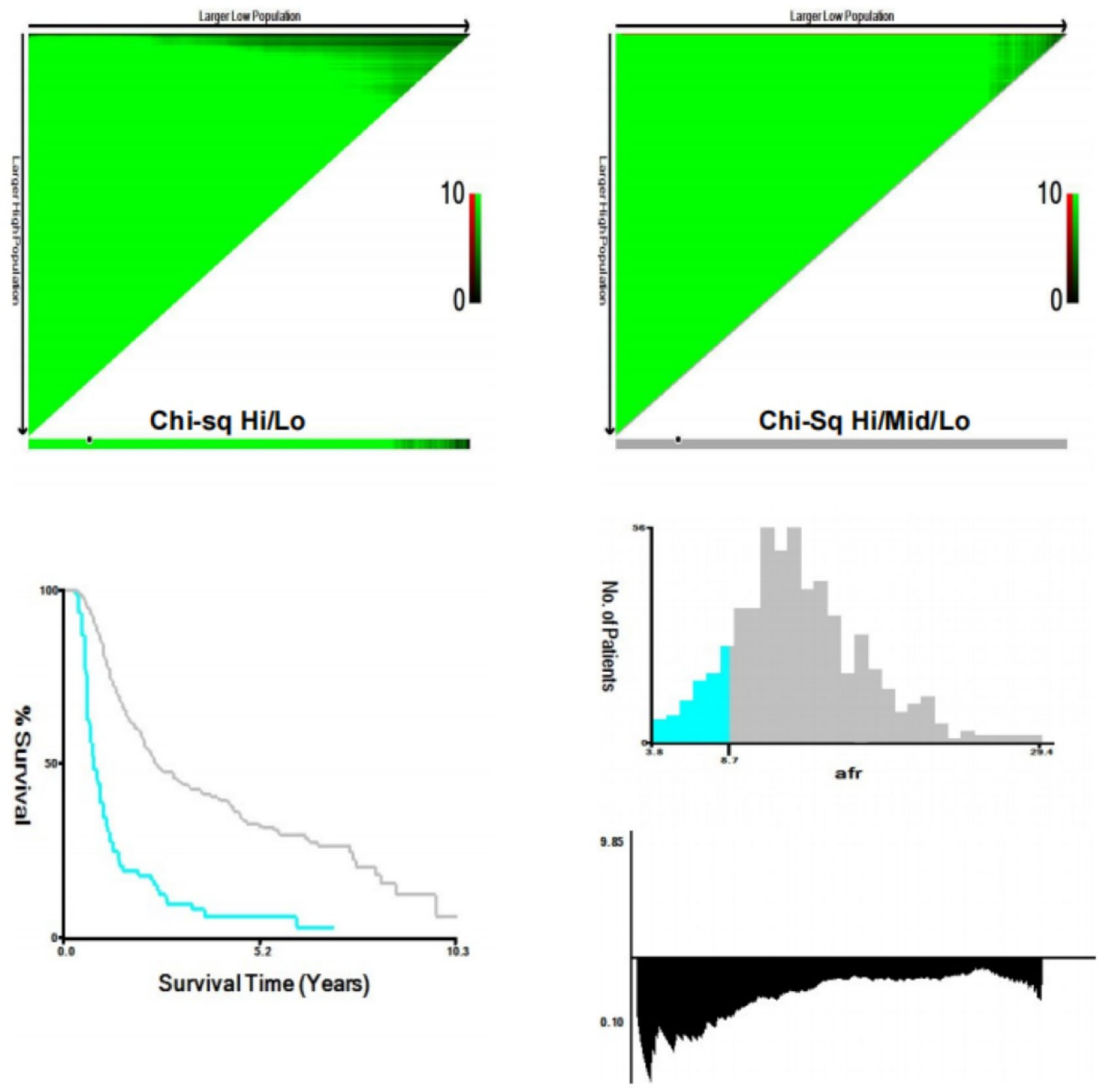
X-tile analyses to determine the optimal cut-off values of AFR. (AFR, albumin-to-fibrinogen ratio).

Continuous variables were expressed as medians with interquartile ranges (IQR), and differences between groups were analyzed using the Mann-Whitney U test. Categorical variables were presented as counts and percentages, with group differences analyzed using Pearson’s chi-square test or Fisher’s exact probability test. The optimal cut-off value (8.71) of the AFR was determined via X-tile (24) (**Figure 2**), categorizing patients into high AFR and low AFR groups. Propensity score matching (PSM) was employed to balance the bias between the two groups, selecting matching variables from baseline characteristics with statistically significant differences,while excluding ALB and FIB (25). Patients were matched in a 1:3 ratio based on propensity score, with a caliper value set at 0.2. Survival analysis was performed using the Kaplan-Meier method, with inter-group differences compared using the log-rank test. Cox regression models were utilized to determine independent prognostic factors for survival in ICC patients. Specifically, variables demonstrating statistical significance (P<0.05) in univariate analysis were simultaneously entered into multivariable Cox models using the enter method. Variables that remained statistically significant (P<0.05) were retained as independent prognostic factors, and nomograms were developed to predict the survival outcomes based on these factors. The concordance index (C-index) (26), decision curve analysis (DCA) (27), calibration curve (28) and area under the receiver operating characteristic (ROC) curve (AUC) (29) were employed to evaluate the clinical validity of the nomogram and to compare its prognostic value with that of other factors and TNM stage.

The related statistical analysis was conducted using SPSS (version 25.0; IBM Corporation, Armonk, NY, USA), RStudio (version 4.4.1, R Foundation for Statistical Computing, Vienna, Austria), and X-tile (version 3.6.1; Yale University, New Haven, CT, USA). All tests were two-tailed and a P value < 0.05 was considered statistically significant.

## 3 Results

### 3.1 Characteristics of the entire study population

A total of 610 patients underwent surgery for ICC from July 2009 to October 2022. Among the rolled patients, 30 were lost to follow-up and 21 patients presented incomplete data, resulting in a final cohort of 559 patients with a median follow-up duration of 23.3 months (IQR: 13.0–38.4 months). As shown in **Table 1**, 273 patients (48.8%) were over 60 years, with 261 were male (46.7%). The majority the patients (91.9%) had good liver reserve function, while only 8.1% were classified as Child–Pugh B/C grade. Multiple tumors were present in 128 patients (22.9%), while 351 patients (62.8%) had tumors with a maximum diameter exceeding 5 cm.

**Table 1:** Clinical and pathologic characteristics of patients with ICC stratified by AFR before and after PSM.

Variables	Before PSM			After PSM		
	Low AFR (n=78)	High AFR (n=481)	*P* value	Low AFR (n=64)	High AFR (n=145)	*P* value
Gender			0.729			0.534
Male	35 (44.9)	226 (47.0)		31 (48.4)	77 (53.1)	
Female	43 (55.1)	255 (53.0)		33 (51.6)	68 (46.9)	
Age			0.231			0.420
≤ 60	35 (44.9)	251 (52.2)		31 (48.4)	79 (54.5)	
> 60	43 (55.1)	230 (47.8)		33 (51.6)	66 (45.5)	
Alcohol history			0.437			0.975
Yes	22 (28.2)	116 (24.1)		14 (21.9)	32 (22.1)	
No	56 (71.8)	365 (75.9)		50 (78.1)	113 (77.9)	
Hypertension			0.857			0.854
Yes	20 (25.6)	128 (26.6)		16 (25.0)	38 (26.2)	
No	58 (74.4)	353 (73.4)		48 (75.0)	107 (73.8)	
Diabetes			0.464			0.325
Yes	9 (11.5)	43 (8.9)		7 (10.9)	10 (6.9)	
No	69 (88.5)	438 (91.1)		57 (89.1)	135 (93.1)	
Hepatitis			0.544			0.293
Yes	19 (24.4)	133 (27.7)		16 (25.0)	27 (18.6)	
No	59 (75.6)	348 (72.3)		48 (75.0)	118 (81.4)	
BMI	23.7 (21.6-26.3)	23.4 (21.2-25.8)	0.752	23.7 (21.7-26.2)	23.4 (21.5-25.8)	0.969
AFP, U/mL	3.2 (1.8-5.0)	3.0 (2.0-5.1)	0.702	3.2 (1.7-5.0)	3.1 (1.9-5.1)	0.673
CEA, ng/mL	3.7 (1.9-13.6)	1.6 (2.7-4.6)	**0.003**	4.7 (1.7-26.3)	3.3 (1.7-9.2)	0.170
CA125, U/mL	39.4 (23.0-91.2)	16.7 (10.7-31.8)	**<0.001**	41.5 (24.8-95.5)	36.4 (16.9-86.1)	0.161
CA199, U/mL	281.1 (33.2-1000.0)	52.4 (16.2-268.3)	**<0.001**	219.8 (20.5-1000.0)	190.8 (34.1-1000.0)	0.972
AST, U/L	36.0 (23.8-66.0)	28.0 (22.0-36.0)	**<0.001**	35.0 (23.0-54.0)	31.0 (23.0-47.0)	0.761
ALT, U/L	29.5 (15.8-90.8)	24.0 (16.0-36.0)	0.064	25.0 (15.0-53.0)	24.0 (17.0-49.0)	0.853
GGT, U/L	139.5 (80.5-343.8)	62.0 (33.0-130.5)	**<0.001**	121.0 (74.5-236.0)	96.0 (48.0-209.0)	0.058
ALB, g/L	36.1 (33.6-38.8)	42.7 (40.3-45.2)	**<0.001**	36.3 (33.9-39.3)	42.3 (40.0-45.8)	**<0.001**
TBIL, μmol/L	15.5 (11.6-40.2)	14.0 (10.7-18.7)	**0.015**	14.7 (11.2-25.0)	14.6 (10.7-20.0)	0.532
FIB, g/L	5.1 (4.5-5.8)	3.1 (2.6-3.6)	**<0.001**	5.1 (4.4-5.7)	3.5 (3.0-3.8)	**<0.001**
NLR	3.5 (2.5-6.5)	2.5 (1.8-3.4)	**<0.001**	3.3 (2.4-6.0)	3.1 (2.3-4.1)	0.051
PLR	167.3 (126.3-222.1)	115.9 (87.8-154.1)	**<0.001**	165.6 (125.8-202.6)	142.1 (106.6-200.6)	0.055
Child-Pugh			**<0.001**			0.290
A	58 (74.4)	456 (94.8)		52 (81.2)	126 (86.9)	
B/C	20 (25.6)	25 (5.2)		12 (18.8)	19 (13.1)	
TNM staging (8th)			0.105			
I/II	35 (44.9)	170 (35.3)		28 (43.7)	59 (40.7)	
III	43 (55.1)	311 (64.7)		36 (56.3)	86 (59.3)	0.679
Tumor number			0.229			
1	56 (71.8)	375 (78.0)		47 (73.4)	107 (73.8)	
>1	22 (28.2)	106 (22.0)		17 (26.6)	38 (26.2)	0.957
Tumor size			**<0.001**			0.504
≤5	16 (20.5)	192 (39.9)		14 (21.9)	26 (17.9)	
>5	62 (79.5)	289 (60.1)		50 (78.1)	119 (82.1)	
Tumor differentiation			**0.044**			0.953
Well	29 (37.2)	238 (49.5)		24 (37.5)	55 (37.9)	
Moderate/Poor	49 (62.8)	243 (50.5)		40 (62.5)	90 (62.1)	
Satellite lesion			0.879			0.421
Yes	11 (14.1)	71 (14.8)		9 (14.1)	27 (18.6)	
NO	67 (85.9)	410 (85.2)		55 (85.9)	118 (81.4)	
MCI			0.182			0.957
Yes	20 (25.6)	92 (19.1)		17 (26.6)	38 (26.2)	
NO	58 (74.4)	389 (80.9)		47 (73.4)	107 (73.8)	
MVI			0.116			0.959
Yes	11 (14.1)	41 (8.5)		9 (14.1)	20 (13.8)	
NO	67 (85.9)	440 (91.5)		55 (85.9)	125 (86.2)	
Perineural invasion			0.263			0.992
Yes	15 (19.2)	69 (14.3)		11 (17.2)	25 (17.2)	
NO	63 (80.8)	412 (85.7)		53 (82.8)	120 (82.8)	
Liver capsule invasion			**0.011**			0.865
Yes	30 (38.5)	260 (54.1)		27 (42.2)	63 (43.4)	
NO	48 (61.5)	221 (45.9)		37 (57.9)	82 (56.6)	
Lymph node metastasis			0.077			0.693
Yes	23 (29.5)	99 (20.6)		18 (28.1)	37 (25.5)	
NO	55 (70.5)	382 (79.4)		46 (71.9)	108 (74.5)	

Data are presented as n (%) or median (IQR); Bold text hinted that these variables were statistically significant.

ICC, intrahepatic cholangiocarcinoma; AFR, albumin-to-fibrinogen ratio; PSM, propensity score matching; BMI, body mass index; AFP, alpha-fetoprotein; CEA, carcinoembryonic antigen; CA125, carbohydrate antigen125; CA19-9, carbohydrate antigen19-9; AST, aspartate aminotransferase; ALT, alanine aminotransferase; GGT, gamma-glutamyltransferase; ALB, albumin; TBIL, total bilirubin; FIB, fibrinogen; NLR, neutrophil to lymphocyte ratio; PLR, platelet to lymphocyte ratio; AJCC, American Joint Committee on Cancer; MCI, macrovascular invasion; MVI, microvascular invasion; IQR, interquartile range.

Spearman’s rank correlation analysis showed a significant negative correlation between AFR and Child-Pugh scores (r = −0.243, P < 0.001).

The optimal cut-off value for AFR was determined to be 8.71 using X-tile software. Based on this threshold, 78 patients (14.0%) were classified into the low AFR group, while 481 patients (86.0%) were placed in the high AFR group. Compared to high AFR group, patients with low AFR exhibited poorer preoperative laboratory test results, including CEA, CA125, CA199, AST, GGT, ALB, TBIL, FIB, NLR and PLR, all P < 0.05). Additionally, they demonstrated worse preoperative status as indicated by the Child-Pugh score (P < 0.001), with a significant negative correlation between AFR and Child-Pugh grade (r = -0.243, P < 0.001), as well as unfavorable oncological characteristics, such as tumor size, tumor differentiation and liver capsule invasion (all P < 0.05). Following a 1:3 matching process, 64 patients (30.6%) were included in the low AFR group and 145 (69.4%) patients were included in the high AFR group, resulting in fully balanced data between the two groups (**Table 1**).

### 3.2 Comparison of OS and DFS based on the AFR before and after PSM

**Figure 3:**
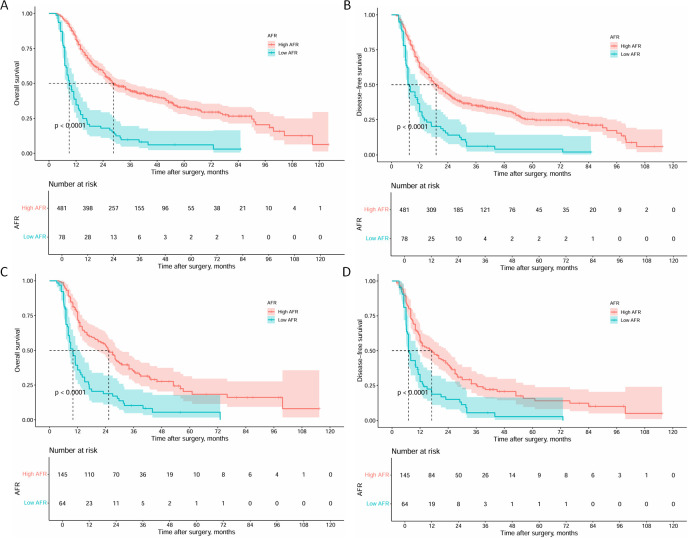
Kaplan-Meier curves of OS and DFS for ICC patients before and after PSM. **(A)** Kaplan-Meier curves of OS for ICC patients before PSM; **(B)** Kaplan-Meier curves of DFS for ICC patients before PSM; **(C)** Kaplan-Meier curves of OS for ICC patients after PSM; **(D)** Kaplan-Meier curves of DFS for ICC patients after PSM. (OS, overall survival; DFS, disease-free survival; ICC, intrahepatic cholangiocarcinoma; PSM, propensity score matching).

Survival analysis of the two cohorts demonstrated distinct outcomes both before and after PSM. Before PSM, the high AFR group exhibited superior survival outcomes, with1 -, 3 -, 5-year OS rates of 81.2%, 44.8% and 32.9% (median: 28.7 months), respectively, compared to 34.6%, 9.7% and 6.1% (median: 9.0 months) in the low AFR group (all *P* < 0.001, **Figure 3A**). DFS rates were 62.6%, 35.1% and 25.3% (median: 19.0 months) in the high AFR group, versus 30.6%, 6.2% and 4.1% (median: 7.6 months) in the low AFR group (all *P* < 0.001, **Figure 3B**).

After PSM, the high AFR group maintained significantly better outcomes. The 1 -, 3 -, and 5-year OS rates were 72.3%, 34.6%and 20.4% (median: 25.3 months) in the high AFR group, compared to 35.9%, 10.2%, and 5.5% (median: 10.0 months) in the low AFR group (all *P* < 0.001, **Figure 3C**). DFS rates followed a similar trend, with 57.0%, 25.2% and 15.7% (median: 17.0 months) in the high AFR group, compared to 27.7%, 5.6%, and 2.8% (median: 7.0 months) in the low AFR group (all *P* < 0.001, **Figure 3D**).

### 3.3 Prognostic factors for OS and DFS

Univariate analysis identified prognostic factors associated with OS, including CEA, CA125, CA199, AFR, AST, GGT, TBIL, NLR, PLR, Child-Pugh, tumor number, tumor size, tumor differentiation, satellite lesion, MCI, perineural invasion and lymph node metastasis (all *P* < 0.05). Further multifactorial analysis revealed CA199 (hazard ratio [HR] 2.146; 95% CI 1.607-2.867; *P* < 0.001), AFR (HR 0.393; 95% CI 0.276-0.560; *P* < 0.001), satellite lesion (HR 1.489; 95% CI 1.063-2.084; *P* = 0.022), and lymph node metastasis (HR 1.638; 95% CI 1.231-2.181; *P* < 0.001) were identified as independent prognostic factors for OS (**Table 2**).

**Table 2:** Univariate and multivariate COX regression analysis for OS of patients with ICC.

Variables	Univariate analysis		Multivariate analysis	
	HR (95% CI)	*P* value	HR (95% CI)	*P* value
Gender, male vs. female	1.120 (0.911-1.377)	0.281		
Age, >60 vs. ≤60, years	1.062 (0.864-1.304)	0.570		
Alcohol, yes vs. no	1.150 (0.909-1.455)	0.243		
Hypertension, yes vs. no	0.972 (0.765-1.235)	0.817		
Diabetes, yes vs. no	1.024 (0.716-1.466)	0.895		
Hepatitis, yes vs. no	1.007 (0.801-1.266)	0.953		
BMI	0.822 (0.639-1.058)	0.128		
CEA, >10 vs. ≤10, ng/mL	2.182 (1.662-2.865)	**<0.001**	1.217 (0.864-1.714)	0.261
CA125, >39 vs. ≤39, U/mL	2.069 (1.594-2.685)	**<0.001**	1.325 (0.986-1.780)	0.062
CA199, >39 vs. ≤39, U/mL	2.445 (1.954-3.060)	**<0.001**	2.146 (1.607-2.867)	**<0.001**
AFR	0.281 (0.216-0.366)	**<0.001**	0.393 (0.276-0.560)	**<0.001**
AST, >40 vs. ≤40, U/L	1.427 (1.134-1.797)	**0.002**	1.223 (0.880-1.699)	0.230
ALT, >50 vs. ≤50, U/L	1.263 (0.983-1.635)	0.068		
GGT, >60 vs. ≤60, U/L	1.582 (1.279-1.957)	**<0.001**	0.993 (0.745-1.324)	0.963
TBIL, >23.5 vs. ≤23.5, μmol/L	1.378 (1.055-1.800)	**0.019**	0.946 (0.607-1.475)	0.806
NLR	1.024 (1.002-1.046)	**0.032**	0.981 (0.939-1.025)	0.397
PLR	1.002 (1.001-1.003)	**<0.001**	1.002 (1.000-1.003)	0.095
Child-Pugh, B/C vs. A	1.526 (1.076-2.163)	**0.018**	0.912 (0.535-1.557)	0.737
TNM staging (8th), III vs. I/II	1.145 (0.924-1.419)	0.105		
Tumor number, >1 vs. 1	1.323 (1.042-1.679)	**0.022**	1.067 (0.789-1.442)	0.673
Tumor size, >5 vs. ≤5	1.323 (1.064-1.645)	**0.012**	1.036 (0.782-1.371)	0.807
Tumor differentiation, Moderate/Poor vs. Well	1.300 (1.056-1.599)	**0.013**	1.105 (0.855-1.427)	0.446
Satellite lesion, yes vs. no	1.886 (1.443-2.464)	**<0.001**	1.489 (1.063-2.084)	**0.022**
MCI, yes vs. no	1.523 (1.182-1.962)	**0.001**	1.178 (0.858-1.616)	0.311
MVI, yes vs. no	1.362 (0.984-1.885)	0.063		
Perineural invasion, yes vs. no	1.381 (1.037-1.840)	**0.027**	0.779 (0.536-1.131)	0.189
Liver capsule invasion, yes vs. no	0.972 (0.791-1.195)	0.790		
Lymph node metastasis, yes vs. no	1.985 (1.567-2.513)	**<0.001**	1.638 (1.231-2.181)	**<0.001**

Data are presented as n (%) or median (IQR); Bold text hinted that these variables were statistically significant.

OS, overall survival; ICC, intrahepatic cholangiocarcinoma; AFR, albumin-to-fibrinogen ratio; PSM, propensity score matching; BMI, body mass index; AFP, alpha-fetoprotein; CEA, carcinoembryonic antigen; CA125, carbohydrate antigen125; CA19-9, carbohydrate antigen19-9; AST, aspartate aminotransferase; ALT, alanine aminotransferase; GGT, gamma-glutamyltransferase; TBIL, total bilirubin; NLR, neutrophil to lymphocyte ratio; PLR, platelet to lymphocyte ratio; AJCC, American Joint Committee on Cancer; MCI, macrovascular invasion; MVI, microvascular invasion; IQR, interquartile range.

In the exploration of prognostic factors for DFS, we found that CEA, CA125, CA199, AFR, GGT, PLR, TNM staging (8th), tumor number, tumor size, tumor differentiation, satellite lesion, MCI, MVI, perineural invasion and lymph node metastasis were significantly associated with DFS (all *P* < 0.05). After adjusting for potential confounding factors in the multifactorial analysis, CA199 (HR 1.690; 95% CI 1.288-2.218; *P*<0.001), AFR (HR 0.538; 95% CI 0.382-0.757; *P*<0.001), satellite lesion (HR 1.528; 95% CI 1.103-2.117; *P* = 0.022), MCI (HR 1.690; 95% CI 1.167-2.108; *P* = 0.003), and lymph node metastasis (HR 1.453; 95% CI 1.080-1.955; *P* = 0.013) remained significantly associated with DFS (**Table 3**).

**Table 3:** Univariate and multivariate COX regression analysis for DFS of patients with ICC.

Variables	Univariate analysis		Multivariate analysis	
	HR (95% CI)	*P* value	HR (95% CI)	*P* value
Gender, male vs. female	1.099 (0.903-1.337)	0.345		
Age, >60 vs. ≤60, years	1.004 (0.825-1.221)	0.971		
Alcohol, yes vs. no	0.960 (0.764-1.206)	0.726		
Hypertension, yes vs. no	1.074 (0.859-1.344)	0.530		
Diabetes, yes vs. no	0.890 (0.626-1.265)	0.517		
Hepatitis, yes vs. no	1.050 (0.844-1.305)	0.662		
BMI	0.882 (0.695-1.118)	0.300		
CEA, >10 vs. ≤10, ng/mL	1.920 (1.477-2.495)	**<0.001**	1.175 (0.840-1.645)	0.346
CA12-5, >39 vs. ≤39, U/mL	1.724 (1.338-2.221)	**<0.001**	1.170 (0.873-1.567)	0.292
CA19-9, >39 vs. ≤39, U/mL	1.821 (1.482-2.238)	**<0.001**	1.690 (1.288-2.218)	**<0.001**
AFR	0.403 (0.310-0.524)	**<0.001**	0.538 (0.382-0.757)	**<0.001**
AST, >40 vs. ≤40, U/L	1.209 (0.967-1.511)	0.096		
ALT, >50 vs. ≤50, U/L	0.999 (0.778-1.282)	0.992		
GGT, >60 vs. ≤60, U/L	1.447 (1.184-1.769)	**<0.001**	0.946 (0.726-1.233)	0.659
TBIL, >23.5 vs. ≤23.5, μmol/L	1.109 (0.851-1.444)	0.445		
NLR	1.016 (0.995-1.038)	0.139		
PLR	1.001 (1.000-1.002)	**0.014**	1.001 (0.999-1.002)	0.235
Child-Pugh, B/C vs. A	1.193 (0.843-1.688)	0.320		
TNM staging (8th), III vs. I/II	1.246 (1.015-1.529)	**0.036**	0.891 (0.676-1.173)	0.410
Tumor number, >1 vs. 1	1.530 (1.224-1.913)	**<0.001**	1.156 (0.865-1.546)	0.327
Tumor size, >5 vs. ≤5	1.285 (1.046-1.580)	**0.017**	1.034 (0.793-1.347)	0.805
Tumor differentiation, Moderate/Poor vs. Well	1.256 (1.033-1.529)	**0.023**	1.099 (0.861-1.403)	0.449
Satellite lesion, yes vs. no	1.901 (1.465-2.467)	**<0.001**	1.528 (1.103-2.117)	**0.011**
MCI, yes vs. no	1.760 (1.388-2.231)	**<0.001**	1.568 (1.167-2.108)	**0.003**
MVI, yes vs. no	1.422 (1.039-1.947)	**0.028**	0.946 (0.634-1.411)	0.785
Perineural invasion, yes vs. no	1.310 (1.000-1.716)	**0.050**	0.846 (0.597-1.199)	0.347
Liver capsule invasion, yes vs. no	0.974 (0.801-1.184)	0.792		
Lymph node metastasis, yes vs. no	1.826 (1.453-2.295)	**<0.001**	1.453 (1.080-1.955)	**0.013**

Data are presented as n (%) or median (IQR); Bold text hinted that these variables were statistically significant.

DFS, disease-free survival; ICC, intrahepatic cholangiocarcinoma; AFR, albumin-to-fibrinogen ratio; PSM, propensity score matching; BMI, body mass index; AFP, alpha-fetoprotein; CEA, carcinoembryonic antigen; CA125, carbohydrate antigen125; CA19-9, carbohydrate antigen19-9; AST, aspartate aminotransferase; ALT, alanine aminotransferase; GGT, gamma-glutamyltransferase; TBIL, total bilirubin; NLR, neutrophil to lymphocyte ratio; PLR, platelet to lymphocyte ratio; AJCC, American Joint Committee on Cancer; MCI, macrovascular invasion; MVI, microvascular invasion; IQR, interquartile range.

### 3.4 Nomogram construction and validation

**Figure 4:**
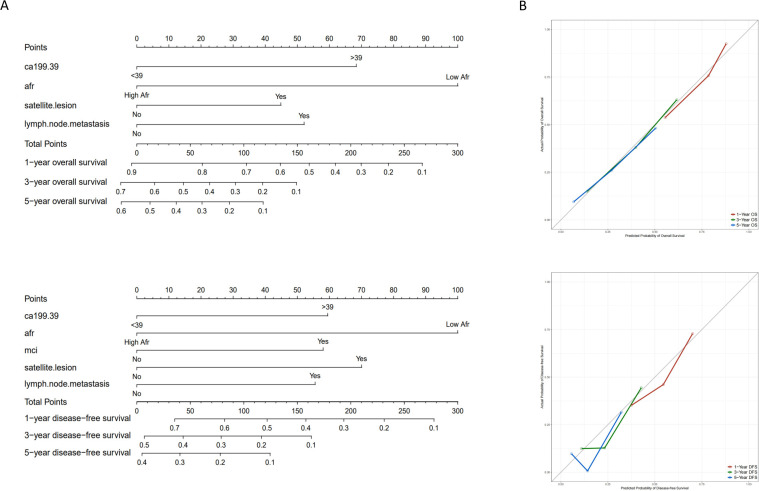
Nomograms and calibration curves for predicting 1-, 3- and 5-year OS and DFS for ICC patients. **(A)** Nomograms for predicting 1-, 3- and 5-year OS and DFS for ICC patients; **(B)** Calibration curves for predicting 1-, 3- and 5-year OS and DFS for ICC patients. (OS, overall survival; DFS, disease-free survival; ICC, intrahepatic cholangiocarcinoma; AFR, albumin-to-fibrinogen ratio; MCI, macrovascular invasion).

**Figure 5:**
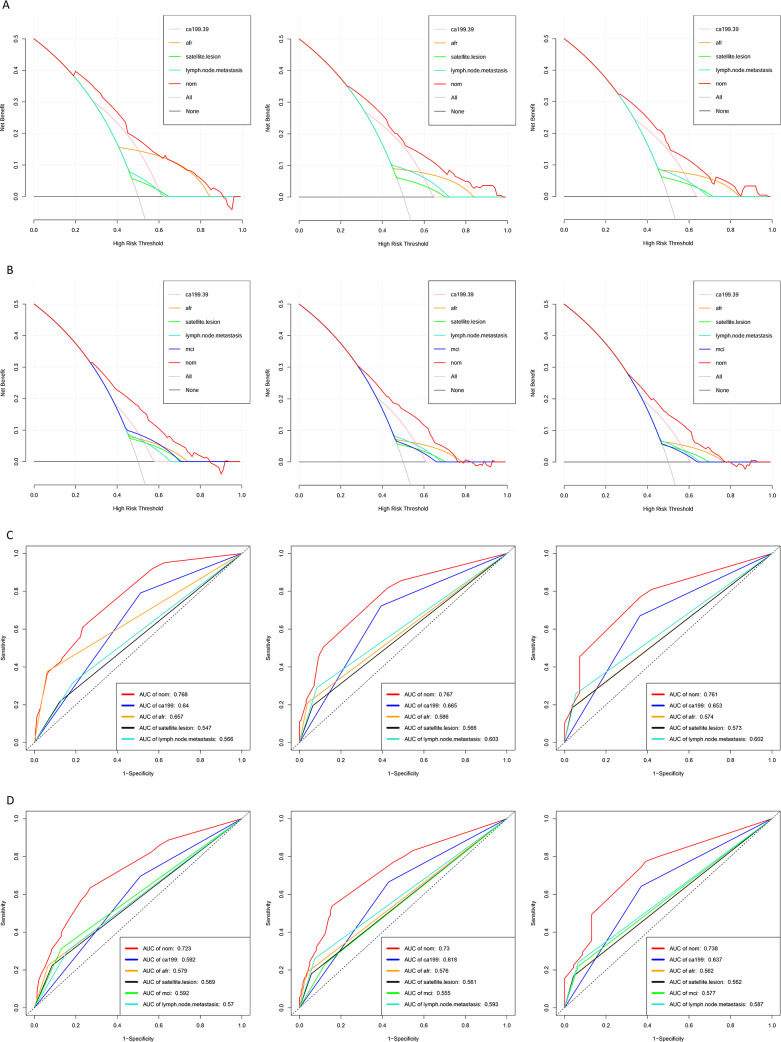
Decision curve analysis and ROC curves for predicting 1-, 3- and 5-year OS and DFS for ICC patients. **(A)** Decision curve analysis for predicting 1-, 3- and 5-year OS for ICC patients; **(B)** Decision curve analysis for predicting 1-, 3- and 5-year DFS for ICC patients; **(C)** ROC curves for predicting 1-, 3- and 5-year OS for ICC patients; **(D)** ROC curves for predicting 1-, 3- and 5-year DFS for ICC patients. (ROC, receiver operating characteristic; OS, overall survival; DFS, disease-free survival; ICC, intrahepatic cholangiocarcinoma; AFR, albumin-to-fibrinogen ratio; MCI, macrovascular invasion; AUC, area under curve).

The prognostic nomograms for OS and DFS were constructed based on the following independent prognostic factors: CA199 (≤39 vs. > 39), AFR (≤8.71 vs. > 8.71), satellite lesion (yes vs. no), lymph node metastasis (yes vs. no) and MCI (yes vs. no) (**Figures 4A, B**). To evaluate the accuracy of the nomograms, the C-index was applied to evaluate the discriminative power of the nomogram against other parameters. For OS prediction, the C-index of the nomogram was 0.700, higher than CA199 (0.617), AFR (0.587), satellite lesion (0.540) and lymph node metastasis (0.562). The C-index for the DFS nomogram was 0.659, also exceeding that of the other parameters. In addition, the calibration curves revealed a relatively accurate agreement between the OS and DFS rates predicted by the nomograms and the actual survival results (**Figures 4C, D**). Finally, as shown in **Figure 5**, the results of DCA and ROC curve indicate that the models have good clinical application value.

### 3.5 Comparison of the predictive value of the nomogram and other factors

The nomogram prediction model was compared with the ROC curve of the 8th edition of the AJCC TNM staging system and five other commonly used prognostic factors for ICC which including Tumor Burden Score (TBS), Prognostic Nutritional Index (PNI), Systemic Inflammatory Index (SII), NLR and PLR. The AUC values of the 1-, 3-, and 5 -year OS nomograms were 0.768, 0.767, and 0.761, respectively, which were significantly greater than the AUC values of TNM (0.526, 0.529, and 0.585, respectively, **Supplementary Figure S1A**) and the five aforementioned prognostic factors. Consistent results were observed for DFS as the nomogram achieved AUC values of 0.723, 0.730, and 0.738 for 1-, 3-, and 5-year DFS, respectively, which were notably superior to the corresponding AUC values of the AJCC 8th edition TNM staging system (0.542, 0.546, and 0.595; **Supplementary Figure S1B**) and the five other prognostic factors.

## 4 Discussion

Although ICC is a relatively rare disease, its incidence and mortality have been increasing worldwide in the past few decades (30, 31). Most patients were diagnosed in advanced stages, resulting a 5-year OS of only approximately 30%, while the recurrence rate is as high as 60%, which seriously affects the quality of life and survival time of patients (32–34). Therefore, identifying preoperative prognostic factors for ICC is highly important for improving patient outcomes.

TNM staging system is valuable for ICC prognosis evaluation. However, owing to the high heterogeneity of ICC, TNM staging cannot fully consider the biological behavior of tumors and other factors, especially in T stage. Guo et al. pointed out that T1 ICC was considered a tumor without vascular invasion, but it usually companied by distant metastasis and thus cannot predict distant metastasis in patients with early-stage tumors (11). Our previous study revealed that the optimal cut-off values of T1 staging for isolated ICC with and without vascular invasion were 8 and 3cm, respectively, which are more helpful in predicting the prognosis of ICC (10). The degree of tumor differentiation can directly reflect the morphological and functional similarity between tumor cells and normal tissue cells, but it is affected by subjective factors, and the boundary is blurred, with an error of approximately 20% (30). CA199 is also a commonly used indicator of the prognosis in ICC patients, but it is not expressed in about 10%-15% of Lewis antigen-negative individuals and is also increased in many benign bile duct diseases such as cholelithiasis and cholangitis (35). Inflammatory markers are closely related to the tumor microenvironment and the prognosis of ICC patients. However, it is susceptible to interference from nontumor factors such as basic diseases and infection (36, 37). As a composite biochemical marker, the AFR is relatively stable in circulation and has been used to evaluate the prognosis of a variety of cancers, including colorectal cancer (38), non-small cell lung cancer (39), and prostate cancer (40).

In ICC patients, ALB levels are often reduced due to tumor-related consumption and impaired liver function, which affects the normal metabolism and function of cells and thereby weakens the body’s ability to surveil tumor cells. Moreover, decreased ALB lowers plasma colloid osmotic pressure, leading to tissue edema and a hypoxic tumor microenvironment. This not only promotes adaptive changes in tumor cells, such as the activation of the hypoxia-inducible factor signaling pathway (41, 42), but also promotes malignant behaviors, including tumor angiogenesis, cell proliferation and invasion, enhancing the cytokine-induced inflammatory cancer-associated fibroblast phenotype (43). In addition, ALB has antioxidant capacity. In the process of tumor development and progression, excessive free radicals are produced, which damaging cell DNA, leading to gene mutations and promoting tumor development (44). Low ALB levels cannot effectively remove free radicals, which exacerbates tumor cells proliferation and progression.

As an important part of the coagulation system, fibrinogen is involved in thrombosis and inflammatory responses (45, 46). In cancer patients, elevated fibrinogen is often associated with a hypercoagulable state and increased thrombotic risk, while abnormal fibrinogen expression and dysfunction are related to tumor cell proliferation, invasion and metastasis. The hypercoagulable state caused by high fibrinogen levels promotes the formation of fibrin clots around tumor cells, which not only provide a physical barrier protecting tumor cells from immune surveillance but also serves as a scaffold for tumor cell adhesion and metastasis (47). Fibrinogen also plays an important role in the inflammatory response. In the tumor microenvironment, it acts as an inflammatory mediator and participates in the recruitment and activation of inflammatory cells. These inflammatory cells release interleukin-6 (IL-6), tumor necrosis factor-α (TNF-α) in tumor tissues, which activate multiple signaling pathways to promote tumor cell proliferation, survival, invasion and metastasis (48, 49).

In tumor patients, AFR comprehensively reflects nutritional status, inflammatory response and coagulation function—distinguishing it from single biomarkers like ALB and NLR, where ALB only mirrors nutritional or hepatic synthetic function and NLR solely captures inflammatory status. This integrated nature enables AFR to play a key role in tumor occurrence, development, and prognosis (50, 51). Notably, the biological basis of the AFR’s prognostic value in ICC differs from that in other cancers, primarily due to this ICC’s unique association with cholestasis and liver fibrosis. In ICC, tumor-related biliary obstruction triggers cholestasis, which activates hepatic inflammation and fibrosis. Activated hepatic stellate cells (HSCs) upregulate fibrinogen synthesis through TGF-β/Smad signaling, leading to fibrinogen elevation (52, 53). This mechanism is less prominent in cancers not involving the biliary-liver axis, such as pancreatic cancer where fibrinogen changes are primarily driven by systemic inflammation. Concurrently, liver fibrosis and cholestasis impair albumin synthesis, further reducing AFR. This dual mechanism, involving cholestasis and fibrosis-driven fibrinogen elevation alongside liver dysfunction-induced albumin reduction, endows AFR with ICC-specific prognostic relevance, allowing it to more accurately reflect the tumor’s interplay with hepatic physiology than in other malignancies. Our Spearman correlation analysis confirmed a strong negative association between AFR and Child-Pugh scores (r=−0.243, P<0.001), solidifying AFR’s role in liver function assessment. Unlike the Child-Pugh score, which relies on subjective clinical indicators such as ascites and isolated biomarkers, AFR integrates ALB—a key Child-Pugh score component, with FIB, which serves as a sensitive marker of hepatic synthetic function and fibrosis. This lets AFR capture subtle liver function changes in ICC patients, such as identifying subclinical impairment in low-AFR Child-Pugh A patients who will face higher postoperative mortality and recurrence risks. Thus, AFR is both an independent prognostic factor and a complement to conventional liver function assessments, guiding personalized care. In this study, we determined the optimal AFR cut-off value (8.71) using X-tile analysis and applied PSM to balance confounding factors between the two groups. Our results showed that the AFR is closely associated to the clinicopathological characteristics and prognosis of ICC patients. Patients with low AFR tended to have poorer liver function (high AST, GGT, and TBIL levels, high Child-Pugh scores, and low albumin levels), evaluated tumor marker levels (CEA, CA199, and CA125) and worse tumor conditions (larger tumor volume, poor tumor differentiation, and liver capsule invasion). Survival analysis revealed that OS and DFS were significantly lower in the low AFR group than in the high AFR group (all *P*<0.001). Additionally, univariate and multivariate Cox regression analyses both identified the AFR as an independent prognostic factor for OS and DFS. Based on the independent prognostic factors for OS and DFS, we constructed nomograms and comprehensively validated their performance. The C index, calibration curve and DCA all revealed that the nomogram outperformed other individual factors in predicting prognosis in almost all ranges and confirmed its clinical utility. ROC curve analysis further confirms that the nomogram provides more accurate prognostic stratification for both OS and DFS compared to other ICC prognostic indicators, including the AJCC 8th edition TNM staging system, TBS, PNI, SII, NLR and PLR. We therefore believe that our nomogram compensates for the limitations of AJCC 8th edition TNM staging system and other widely used prognostic factors, thereby providing a more accurate prediction of prognosis for ICC patients.

Notably, AFR is an inexpensive and easily obtained biomarker, making it highly practical for routine clinical practice. Unlike complex multi-parameter models that often require specialized tests or multiple procedural steps, AFR is derived exclusively from two routine preoperative blood tests, and this reliance on ubiquitous laboratory data eliminates the need for additional invasive procedures or costly analyses, ensures results are typically available within hours, and grants the marker broad applicability even in resource-limited settings where advanced testing is unavailable.

However, the study still has several limitations. First, this is a retrospective study, selection bias is inevitable. Second, AFR was measured only once using a single preoperative blood sample, which cannot fully reveal the dynamic changes throughout the disease course. In future studies, we will incorporate serial AFR measurements during follow-up to further refine its clinical utility as a dynamic monitoring tool. Third, the current study was conducted in Chinese hospitals, with most patients of Chinese ethnicity and a relatively high HBV infection rate, which limits its generalizability of our finding to other populations. In addition, the clinical utility of AFR was not evaluated in the context of contemporary ICC treatments, including patients receiving targeted therapies like FGFR inhibitors or immune checkpoint blockade. Therefore, future international multicenter studies are required to validate the global applicability of AFR and our nomograms, and prospective studies should further systematically evaluate AFR in combination with molecular profiling to determine its role in predicting response to specific therapeutic modalities.

## 5 Conclusion

The preoperative AFR is an independent prognostic factor for postoperative OS and DFS in ICC patients, and higher levels of AFR predict better OS and DFS. The nomograms including the AFR provide good prognostic prediction for ICC patients after surgery. On the basis of its availability and low cost, personalized treatment strategies based on the AFR are expected to be developed in the future. In future studies, in addition to prospective studies measuring the AFR at multiple time points, larger scale and multicenter clinical studies can be carried out to further verify the role of the AFR in guiding the treatment decisions of ICC patients.

## Data Availability

The raw data supporting the conclusions of this article will be made available by the authors, without undue reservation.

## Supplementary material

Supplementary Figure S1 ROC curves comparing the nomogram and other factors for predicting 1-, 3- and 5-year OS and DFS in ICC patients. **(A)** ROC curves for predicting 1-, 3- and 5-year OS for ICC patients; **(B)** ROC curves for predicting 1-, 3- and 5-year DFS for ICC patients. (ROC, receiver operating characteristic; OS, overall survival; DFS, disease-free survival; ICC, intrahepatic cholangiocarcinoma; TBS, tumor burden score; PNI, prognostic nutritional index; SII, systemic inflammatory index; NLR, neutrophil-to-lymphocyte ratio; PLR, platelet-to-lymphocyte ratio; AUC, area under curve).
