# Supplementary figures and images for: The prognostic value of the preoperative albumin-to-fibrinogen ratio in patients with intrahepatic cholangiocarcinoma: a multicenter retrospective propensity score matching analysis
Source: Front Oncol. 2025 Nov 3;15:1633488. doi: 10.3389/fonc.2025.1633488 (PMC12620261; doi:10.3389/fonc.2025.1633488)

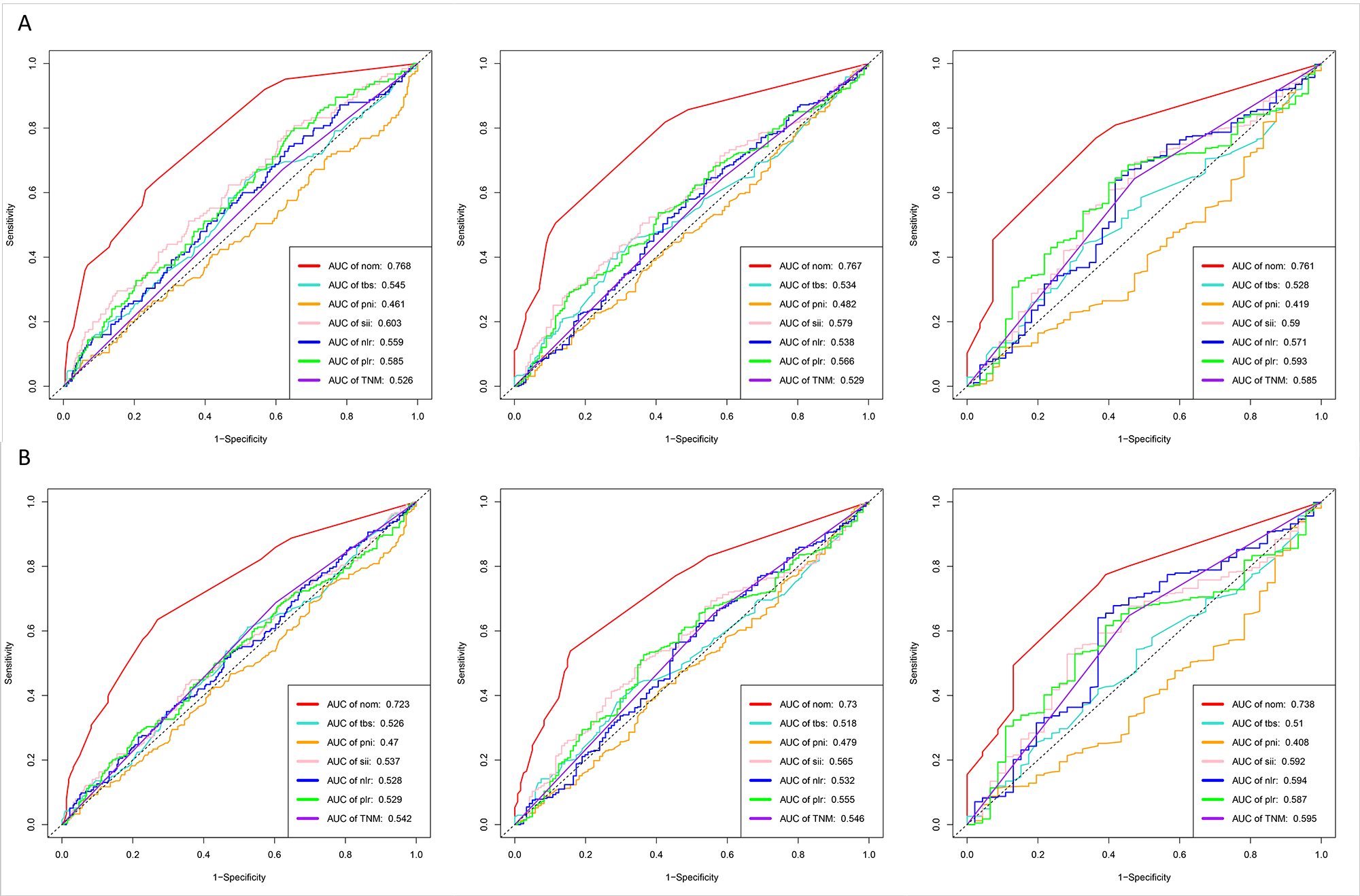

Supplement: Supplementary Figure S1 — ROC curves comparing the nomogram and other factors for predicting 1-, 3- and 5-year OS and DFS in ICC patients. (A) ROC curves for predicting 1-, 3- and 5-year OS for ICC patients; (B) ROC curves for predicting 1-, 3- and 5-year DFS for ICC patients. (ROC, receiver operating characteristic; OS, overall survival; DFS, disease-free survival; ICC, intrahepatic cholangiocarcinoma; TBS, tumor burden score; PNI, prognostic nutritional index; SII, systemic inflammatory index; NLR, neutrophil-to-lymphocyte ratio; PLR, platelet-to-lymphocyte ratio; AUC, area under curve). [file Image1.tif]
